# Antibiotic Resistance Profiling of Pathogenic *Staphylococcus* Species from Urinary Tract Infection Patients in Benin

**DOI:** 10.1155/2023/6364128

**Published:** 2023-05-15

**Authors:** Funkè F. Assouma, Haziz Sina, Ange D. Dossou, Akim Socohou, Milka C. Hounsou, Patrice H. Avogbe, Bawa Boya, Wassiyath Mousse, Adolphe Adjanohoun, Lamine Baba-Moussa

**Affiliations:** ^1^Laboratory of Biology and Molecular Typing in Microbiology, University of Abomey-Calavi, Benin; ^2^Ministry of Heath, Cotonou, 01, BP 363, Benin; ^3^Laboratory of Biochemistry and Molecular Biology, Faculty of Science and Technology, University of Abomey-Calavi, Benin; ^4^National Agronomic Research Institute of Benin, 01, BP 884 Cotonou, Benin

## Abstract

Staphylococci can cause urinary tract infections (UTIs). These UTIs are among the significant causes of antibiotic resistance and the spread of antibiotic-resistant diseases. The current study is aimed at establishing a resistance profile and determining the pathogenicity of *Staphylococcus* strains isolated from UTI samples collected in Benin. For this purpose, urine samples (one hundred and seventy) that were collected from clinics and hospitals showed UTI in patients admitted/visited in Benin. The biochemical assay method was used to identify *Staphylococcus* spp., and the disk diffusion method tested the antimicrobial susceptibility. The biofilm formation ability of the isolates of *Staphylococcus* spp. was investigated by the colorimetric method. The presence of *mecA*, *edinB*, *edinC*, *cna*, *bbp*, and *ebp* genes was examined by multiplex polymerase chain reaction (PCR). The results showed that *Staphylococcus* species were identified in 15.29% of all infected individuals and that 58% of these strains formed biofilms. Most *Staphylococcus* strains (80.76%) were isolated in female samples, and the age group below 30 years appeared to be the most affected, with a rate of 50%. All *Staphylococcus* strains isolated were 100% resistant to penicillin and oxacillin. The lowest resistance rates were seen with ciprofloxacin (30.8%), gentamicin, and amikacin (26.90%). Amikacin was the best antibiotic against *Staphylococcus* strains isolated from UTIs. The isolates carried *mecA* (42.31%), *bbp* (19.23%), and *ebp* (26.92%) genes in varying proportions. This study provides new information on the risks posed to the population by the overuse of antibiotics. In addition, it will play an essential role in restoring people's public health and controlling the spread of antibiotic resistance in urinary tract infections in Benin.

## 1. Introduction

In clinical settings, the most common infections are urinary tract infections (UTIs) that are the second most common after respiratory tract infections [1]. UTIs affect, worldwide, approximately 150 million people annually [2]. The prevalence of UTIs was 67.96% in Benin, with a predominance of women (62.50%) [3]. However, the etiology of these infections has been extensively studied in recent decades, and only a few bacterial species are considered authentic uropathogens [4]. Identification of bacteria and preparation of antibiograms are routine laboratory procedures [5]. *Enterobacteriaceae* and Gram-positive bacteria are the most isolated infectious agents in UTIs. Bacteria typically responsible for 5–10% of urinary tract infections are *Enterococcus faecalis*, *Streptococcus agalactiae*, and *Staphylococcus* spp. [6–11]. In sexually active women, the second most common cause of uncomplicated UTIs is *Staphylococcus* spp. [12]. The mainly isolated *Staphylococcus* species are *S. saprophyticus*, *S. aureus*, *S. warneri*, *S. epidermidis*, *S. hominis*, *S. lentus*, and *S. haemolyticus* [13]. After identifying pathogens, UTIs are treated with broad-spectrum antibiotics [14]. However, antibiotics' inappropriate use has increased antimicrobial resistance (AMR). Thus, the occurrence of AMR induces a negative course after antibiotic treatment among UTI patients and is responsible for severe clinical complications [15, 16]. Multidrug-resistant of urinary tract infection isolates have been reported in Italy [11]. A study conducted in Afghanistan found increasing antibiotic resistance in staphylococci to ampicillin, amoxicillin, and erythromycin [17].

Strains of *Staphylococcus* spp. have been increasingly isolated from urine specimens, although urine is not a reservoir for staphylococci [18]. This leads to the consideration of staphylococci as urinary pathogens. Staphylococci have a simple technique to respond to the threats of a hostile host that opposes it with antibodies, phagocytosis, or cytotoxicity [19]. Staphylococcal infections rely on the production of surface proteins and toxins. Those toxins are used to (i) initiate adhesion of the bacterium to host tissues, (ii) secrete extracellular toxins and enzymes that destroy the host, (iii) inactivate the host immune system and the growth, and (iv) spread the bacteria in host cells and tissues [20]. Virulence factors associated with clinical staphylococcal infections include inhibitors of epidermal cell differentiation (*edin*), bacterial collagen adhesins (*cna*), bone-binding proteins (*bbp*), and activators of binding proteins (*ebp*). In addition, *Staphylococcus* spp. can form biofilms, which increase their virulence and antibiotic tolerance by a factor of 100 to 1000 compared with isolates that do not form biofilm [21]. Biofilm confers antibiotic resistance through processes including encoding antibiotic resistance genes, antibiotics, and even overcoming host immunity [22].

Due to the rapid increase in bacteria's adaptive strategies, a significant change in the etiology and antibiotic resistance profile of uropathogens bacterial has been noted [7, 13]. The presence of *Staphylococcus* strains in urine, in conjunction with their resistance to antibiotics, threatens the prognosis of human life and poses a public health problem. New diagnostic and therapeutic strategies are needed for better treatment of urinary tract infections [23]. Against this background, the present study is aimed at drawing the antibiotic resistance profile and evaluating the virulence potential of staphylococci strains isolated from urine samples collected in Benin.

## 2. Material and Methods

### 2.1. Sampling

The formula from Schwartz [24]: *n* = *t*^2^ × *p*(1 − *p*)/*m*^2^ was used to determine the sample size (*n* is the sample size, *t* is the 95% confidence level, *p* is the prevalence of urinary tract infections (11.7%) [25], and *m* is the 5% margin of error (typical value 0.05). Thus, 170 urine samples were collected from different health facilities (“Bon Samaritain” Clinic of Porto-Novo, Borgou/Alibori Departmental and University Hospital Center, Ouémé/Plateau Departmental and University Hospital Center, Mono/Couffo Departmental Hospital Center, Zou/Collines Departmental Hospital Center, Area Hospital of Djougou, Area Hospital of Ménontin, and Area Hospital of Natitingou) in Benin with sterile wide-mouthed urine cups. These specimens were collected from hospitalized and nonhospitalized patients complaining of urinary tract infections from 05 January 2021 to 30 October 2021. The request form completed by the physicians was used as standard protocol to document the sociodemographic data of study participants. Inclusion criteria were as follows: (i) participation agreement, (ii) presumed diagnosis of UTI, and (iii) no antibacterial therapy two weeks before participation.

### 2.2. *Staphylococcus* Strains' Isolation and Identification

After collection, samples were subjected to standard microbiological tests, including fresh state and Gram stain [26]. The culture was then established on Chapman at 37°C for 24 hours, and colonies suspected to be *Staphylococcus* spp. were confirmed by DNase assay, catalase assay, and the use of API® Staph (bioMérieux, France) [26]. The isolated *Staphylococcus* spp. was confirmed by molecular biology using classical polymerase chain reaction (PCR) [27].

### 2.3. Sensitivity of Isolates to 8 Antibiotics

The sensitivity of the isolated staphylococci to 8 antibiotics was evaluated by the Mueller-Hinton agar medium disk diffusion method following the recommendations of the antibiotic commission of the French Society of Microbiology with McFarland 0.5 as standard control [28]. Penicillin G (P-6 *μ*g), oxacillin (OXA-5 mg), clotrimazole (CLT 50 *μ*g), gentamicin (GM-15 mg), ciprofloxacin (CIP-5 mg), amikacin (AN-30 UI), vancomycin (VA-30 mg), and Augmentin (AMC 20/10 *μ*g) were the eight antibiotics used in this study.

### 2.4. Bacterial Ability to Form Biofilms

We used a qualitative assay to detect biofilm-producing *Staphylococcus* isolates [29, 30]. The biofilm formation based on the appearance of a visible film was accessed *in vitro* on a microplate. Thus, from an 18-hour culture in Brain Heart Infusion, a microplate was inoculated with 10 *μ*l of bacterial suspension supplemented with 150 *μ*l of Brain Heart Infusion. The microplates were incubated at 37°C for 24 hours and then washed thrice to eliminate free bacteria. Crystal violet (0.1%) was used to stain for 10 minutes sessile organisms to the polystyrene support in each of the wells. An additional thorough washing with sterile distilled water was performed to remove excess stain, and the plates were dried at room temperature [31]. The appearance, after air-drying, of a visible film on the walls of the microplate indicated the formation of a biofilm.

### 2.5. Molecular Identification of Virulence Factors

Virulence factors were identified by the molecular method of multiplex PCR (3Prime Thermal Cycler) on extracted DNA using specific primers (Table 1). DNA extraction and amplifications were carried out using the technic of Socohou et al. [30]. The obtained amplicons were separated using agarose gel electrophoresis in 1x TBE buffer with ethidium bromide. After migration at 100 V for 30 minutes, gels were visualized with a UV table. The genes sought were *mecA*, *edinB*, *edinC*, *can*, *ebp*, and *bbp*.

### 2.6. Data Analysis

Data were entered and analyzed using the MS Excel 2013 spreadsheet. The resistance percentage was calculated for each. R 4.2.1 software was used to analyze the resistant and biofilm-forming *Staphylococcus* species possessing virulence genes. The threshold for statistical significance was set at *p* < 0.05.

## 3. Results

### 3.1. Frequency of Isolation of *Staphylococcus* spp.

**Figure 1:**
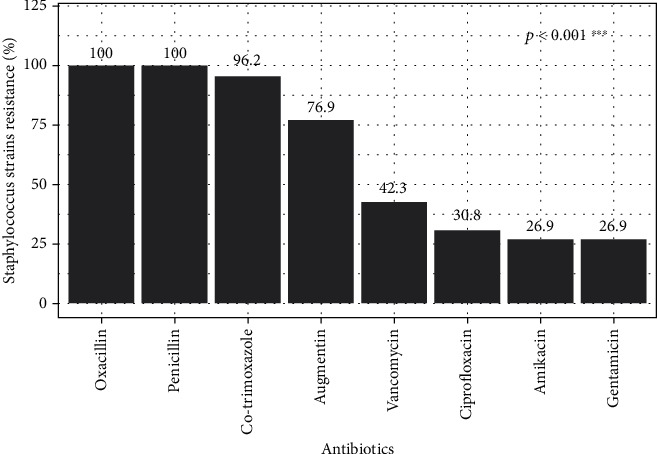
*Staphylococcus* spp. isolates' antibiotic resistance rate.

Of the 170 urine samples included in this study, Gram stain evaluation and biochemical identification revealed that *Staphylococcus* species were found in 15.29% of the cases. Of the *Staphylococcus* spp. positive samples, 80.76% (*n* = 21) were from women. Analysis of Table 2 shows that the 20 to 30 years (50%) age group is the most commonly affected, followed by those between 31 and 40 years (48.15%). Of the isolated staphylococci, 73.07% were coagulase positive and 26.93% were coagulase negative (Figure 1).

### 3.2. Susceptibility to Antibiotics of Strains of *Staphylococcus* spp. Isolated

All isolates of *Staphylococcus* spp. were resistant to penicillin and oxacillin. Resistance was 96.2% to cotrimoxazole and 76.9% to Augmentin. The strains were 42.30% resistant to vancomycin. The lowest resistance was found in ciprofloxacin (30.8%), gentamicin, and amikacin (26.90%), a highly significant difference between the efficacy of antibiotics and UTI strains.

**Figure 2:**
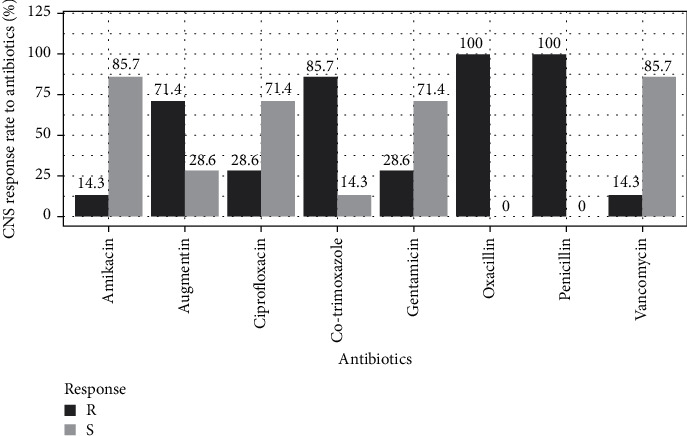
Coagulase-negative staphylococci (CNS) isolates' antibiotic resistance rate.

**Figure 3:**
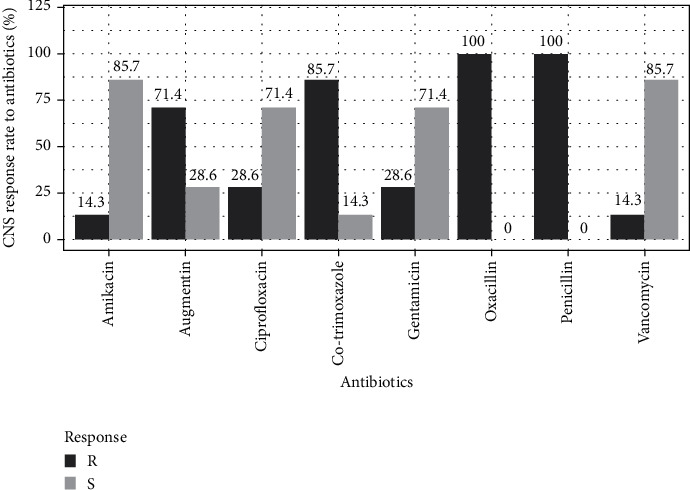
Coagulase-positive staphylococci (CPS) isolates' antibiotic resistance rate.

Regardless of species, antibiotics such as penicillin, oxacillin, cotrimoxazole, and Augmentin were more than 50% resistant. The lowest resistance was observed with vancomycin and amikacin (14.3%) for coagulase-negative staphylococci (CNS) (Figure 2). For *S. aureus*, the least resistance was observed to gentamycin (26.3%), followed by ciprofloxacin (31.6%) (Figure 3).

### 3.3. Biofilm Formation Ability of Isolated *Staphylococcus* spp.

**Figure 4:**
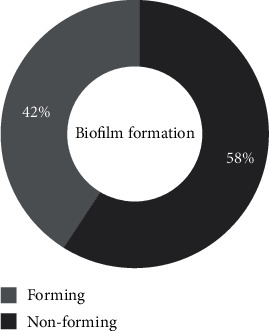
*Staphylococcus* spp. isolates' biofilm production rate.

**Figure 5:**
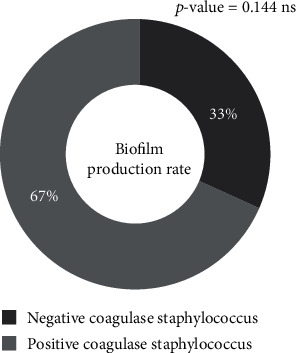
*Staphylococcus* spp. isolates' biofilm production rate according to species.

Figure 4 shows that 58% of *Staphylococcus* spp. isolates formed a bacterial biofilm. *S. aureus* strains formed the most biofilm, with a proportion of 67% (Figure 5). The biofilm formation by isolated species is not statistically significant (*p* = 0.144).

### 3.4. Toxigenic Profile of Isolated *Staphylococcus* spp. Strains

**Figure 6:**
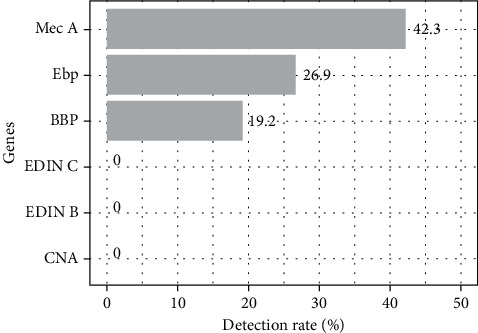
Proportion of genes sought.

**Figure 7:**
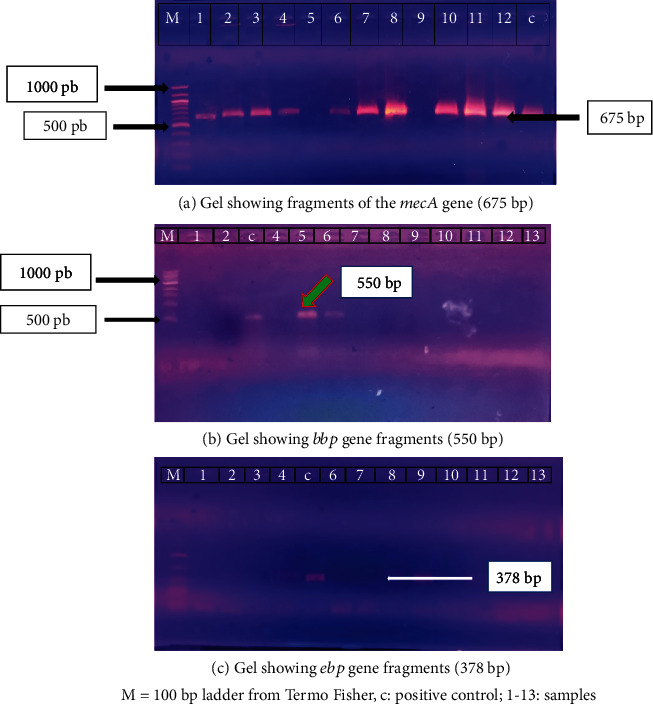
Detection of genes for production of *mecA*, *bbp*, and *ebp* in DNA extracted from *Staphylococcus* strains isolated from urine samples.

**Figure 8:**
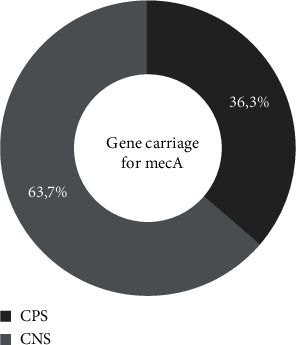
Carrying of genes to mecA.

Figure 6 shows that 42.31% of our strains harbored the gene encoding for methicillin resistance (*mecA*). In addition, among the other studied genes, only those encoding for *bbp* (19.23%) and *ebp* (26.92%) were detected. Gels showing the *mecA*, *bbp*, and *ebp* gene fragments are presented in Figures 7(a)–7(c). Looking at the individual species, we find that 63.7% of CNS possess the *mecA* gene compared to 36.3% in CPS (Figure 8). The *bbp* and *ebp* genes are detected only in CPS.

## 4. Discussion

UTIs are common in hospitals and the community and are caused by Gram-negative and Gram-positive bacteria. Our study shows that 15.2% of urine samples contained strains of *Staphylococcus* spp. This rate remains close to the 16.97% found in a survey conducted in a provincial hospital in northern Morocco [32]. However, lower rates (4.2% to 8.13%) were reported in studies conducted in Rabat [33], in the city of Nouakchott [34], and in Dakar [35]. These variations in the prevalence of *Staphylococcus* strains in urine may be due to the detection methods used in the laboratory and the geographic regions where samples were collected. The high proportion of staphylococci in our study indicates that *Staphylococcus* spp. are more common in the urine, may be considered uropathogenic, and need to be investigated for an excellent treatment strategy for these infections.

Among patients with urinary tract infections caused by *Staphylococcus* spp., we found a significant difference in gender distribution (men 19.24% and women 80.76%). These data are consistent with the epidemiological data reported in Benin [18] and elsewhere [36–38], which show that UTIs are more common in women than in men. There are several reasons for this preponderance of women: the anatomy of the female urinary tract, which consists of a short urethra that does not provide adequate protection against contamination from the vagina and rectum, the imbalance of the saprophyte flora in the urethra and vagina due to excessive hygiene, the prescription of estrogen-progestin treatments, the use of spermicidal gel, and the diaphragm. The effect of prostatic secretions allows for additional protection in men [39].

The age distribution in our study shows that the patients most commonly affected by urinary tract infections caused by *Staphylococcus* spp. are under 30 years old. In the literature, UTI is usually found in older people (>60 years) due to urinary stasis, estrogen deficiency in postmenopausal women, and decreased immune response in the elderly [40].

In our study, 73.07% of isolated staphylococcal strains were positive for coagulase (*Staphylococcus aureus*). This predominance of *Staphylococcus aureus* in urine samples was previously observed in Algeria at 74% [41]. Increased Gram-positive cocci in UTIs have also been reported [42]. In addition, coagulase-negative staphylococci play an essential role in diseases such as UTI [43]. These differences concerning our results could be explained by the timing of the study and sociodemographic parameters. The presence of *S. aureus* in the urine could be defined by its ability to adhere to the extracellular matrix's components and cells, releasing toxins such as enterotoxins, its virulence factors (synthetic bacterial amino acid), which is not usual in urine, and its ability to penetrate from the urethra into the bladder.

The susceptibility of isolates has shown that resistance is present to varying degrees depending on the antibiotic family and the isolated species. *Staphylococcus* strains (coagulase-positive and -negative) show a relatively high resistance rate to penicillin, oxacillin, Augmentin, and cotrimoxazole. This observation is shared by other authors in Benin [3] and Morocco [44]. Coagulase-positive staphylococci (*S. aureus*) strains showed high resistance to vancomycin (52.6%). Compared to the vancomycin resistance rate, our results differ from those obtained in Mauritania and Morocco in *S. aureus*, where vancomycin was more active with a susceptibility rate of over 90% [33, 34]. This difference could be explained by the degree of contact between the strains of *S. aureus* and this antibiotic in our country. Several hypotheses can explain the high antibiotic resistance profiles observed in our study, not only the excessive use of antibiotics in medical care and agriculture but also the lack of regulation of the marketing and acquisition of antibacterial agents. This is compounded by self-medication with often arbitrary dosages due to inadequate regulatory requirements and premature treatment discontinuation as soon as symptoms disappear. Trafficking of smuggled molecules is also a factor in the emergence of antibiotic resistance, with molecules often containing low doses or no active ingredients. Weak resistance of *S. aureus* strains has been recorded with ciprofloxacin, amikacin, and gentamicin. The Gabon study on urine samples showed low resistance rates of *S. aureus* isolates to amikacin and gentamicin [45]. A low resistance rate to ciprofloxacin, amikacin, and vancomycin was recorded in the CNS.

Many staphylococci can form biofilm. In our study, 58% of isolated staphylococcal strains formed biofilm. Our results are lower than the 87.87% obtained in Hungary on clinical strains [46] and the 88.70% obtained in Algeria for staphylococcal strains isolated from human infections, animals, and foods [47]. However, fewer biofilm formations than our results were obtained in Benin for *S. aureus* strains from surfaces and medical devices [30]. This difference could be due to the fact that the formation of biofilm is a complex phenomenon influenced by many factors, including the surrounding environment and genetic regulation factors [48]. Biofilm formation is found in *S. aureus* (67%) and coagulase-negative staphylococci (33%). Our biofilm-forming strains can therefore be considered pathogenic germs, as their virulence also lies in their ability to produce an extracellular matrix and form a biofilm [49]. Forming a biofilm can also hinder the action of antibiotics and lead to the persistence of infections.

Staphylococci possess several factors that contribute to host cell destruction and thus increase the infection risk. Our study of some virulence factors revealed that our staphylococcal strains carried the genes encoding adhesion factors such as *ebp* (26.92%) and *bbp* (19.23%). The presence of these genes encoding adhesion factors at high levels in our isolated strains can be justified by the fact that *S. aureus* is an enteropathogenic bacterium [50]. Our staphylococcal strains' results on the nonproduction of the *edin* B, *edin* C, and *cna* genes are reassuring since the *edin* and *cna* exotoxins are essential virulence factors in host tissue invasion and promoting bacterial colonization during clinical infections.

In our study, 42.31% of *Staphylococcus* strains produced the *mecA* gene. This result is nearly 50.6% of the mecA gene detected in Brazil's *S. aureus* strains isolated from blood culture bottles [51]. Thus, the presence of the gene encoding for *mecA* gives a better idea of the methicillin-resistant strains isolated in our samples.

## 5. Conclusion

This study conducted in Benin provided information on the prevalence of *Staphylococcus* spp. involved in UTIs and their resistance rate to eight antibiotics. Women are most affected by urinary tract infections. Antibiotic resistance is relatively high for molecules such as penicillin, oxacillin, amoxicillin-clavulanic acid, and cotrimoxazole. However, amikacin, gentamicin, and ciprofloxacin showed good antimicrobial activity against *Staphylococcus* isolates in our study. The emergence of *S. aureus* strains with decreased vancomycin sensitivity may pose severe therapeutic problems. Indeed, these data can guide clinicians in choosing a first-line antibiotic. Still, a second antibiogram is necessary to verify the efficacy of the initial treatment and choose the best antibiotics for secondary treatment. These results also highlight the need for new therapeutic agents with new mechanisms of action. Antitoxin therapies aimed at reducing the virulence of bacteria may be a promising alternative. However, some factors determining *Staphylococcus* spp. pathogenicity have been identified; further exploration of virulence factors is a prerequisite for developing a comprehensive understanding of UTI *Staphylococcus* spp.

## Figures and Tables

**Table 1:** Nucleotide sequences and amplification sizes of the genes investigated.

Genes	Sequences	Size
*MecA*	5′-TCCAGGAATGCAGAAAGACC-3′ 5′-TCACCTGTTTGAGGGTGGAT-3.	675 pb
*EdinB*	5′-ATGGTCAAG CCCAGACAGAG-3′ 5′-CGTGTTTTCAACATTTAATGCAA-3′	522 pb
*EdinC*	5′-ACAGTTCAAAAGACAAAGAAGCTATT-3′ 5′-AGGTCTTCCAGCTAATGCAGCTCCTT-3′	543 pb
*Cna*	5′-GTCAAGCAGTTATTAACACCAGAC-3′ 5′-AATCAGTAATTGCACTTTGTCCACTG-3′	278 pb
*Ebp*	5′-CATCCAGAACCAATCGAAGAC-3′ 5′-CTTAACAGTTACATCATCATGTTTATCTTTG3′	378 pb
*Bbp*	5′-AACTACATCTAGTACTCAACAACAG-3′ 5′-ATGTGCTTGAATAACACCATCATCT-3′	550 pb

**Table 2:** Recurrence of *Staphylococcus* spp. urinary tract infections according to age and sex.

Parameter		Proportion
Sex	Female	80.76%
	Male	19.24%

Age	(20-30 years)	50%
	(31-40 years)	46.15%
	(41-50 years)	3.85%

## Data Availability

The data used to support the findings of this work are available from the corresponding author upon request.

## References

[1] Najar M. S., Saldanha C. L., Banday K. A. (2009). Approach to urinary tract infections. *Indian Journal of Nephrology*.

[2] Flores-Mireles A. L., Walker J. N., Caparon M., Hultgren S. J. (2015). Urinary tract infections: epidemiology, mechanisms of infection and treatment options. *Nature Reviews Microbiology*.

[3] Dougnon V., Assogba P., Mohammed J. (2021). Urinary tract infections in Benin: exploring the virulence factors and antibiotic resistance and virulence genes among bacterial isolates. *International Journal of Pathogen Research*.

[4] Kline K. A., Lewis A. L. (2016). Gram-positive uropathogens, polymicrobial urinary tract onfection, and the emerging microbiota of the urinary tract. *Microbiology Spectrum*.

[5] Li W., Sun E., Wang Y. (2019). Rapid identification and antimicrobial susceptibility testing for urinary tract pathogens by direct analysis of urine samples using a MALDI-TOF MS-based combined protocol. *Frontier in Microbiology*.

[6] Gajdács M., Ábrók M., Lázár A., Burián K. (2019). Comparative epidemiology and resistance trends of common urinary pathogens in a tertiary-care hospital: a 10-year surveillance study. *Medicina*.

[7] Manges A. R., Natarajan P., Solberg O. D., Dietrich P. S., Riley L. W. (2006). The changing prevalence of drug-resistant Escherichia coli clonal groups in a community: evidence for community outbreaks of urinary tract infections. *Epidemiology and Infections*.

[8] Akram M., Shahid M., Khan A. U. (2007). Etiology and antibiotic resistance patterns of community-acquired urinary tract infections in J N M C Hospital Aligarh, India. *Annals of Clinical Microbiology and Antimicrobials*.

[9] Akortha E. E., Ibadin K. O. (2008). Incidence and antibiotic susceptibility pattern of *Staphylococcus aureus* amongst patients with urinary tract infection (UTI) in UBTH Benin City, Nigeria. *African Journal of Biotechnology*.

[10] Mirsoleymani S. R., Salimi M., Shareghi B. M., Ranjbar M., Mehtarpoor M. (2014). Bacterial pathogens and antimicrobial resistance patterns in pediatric urinary tract infections: a four-year surveillance study (2009–2012). *International Journal of Pediatrics*.

[11] Serretiello E., Folliero V., Santella B. (2021). Trend of bacterial uropathogens and their susceptibility pattern: study of single academic high-volume center in Italy (2015–2019). *International Journal of Microbiology*.

[12] Argemi X., Hansmann Y., Prola K., Prévost G. (2019). Coagulase-negative staphylococci pathogenomics. *International Journal of Molecular Sciences*.

[13] Bitew A., Molalign T., Chanie M. (2017). Species distribution and antibiotic susceptibility profile of bacterial uropathogens among patients complaining urinary tract infections. *BMC Infectious Diseases*.

[14] Biswas D., Gupta P., Prasad R., Singh V., Arya M., Kumar A. (2006). Choice of antibiotic for empirical therapy of acute cystitis in a setting of high antimicrobial resistance. *Indian Journal of Medical Sciences*.

[15] Franci G., Folliero V., Cammarota M. (2018). Epigenetic modulator UVI5008 inhibits MRSA by interfering with bacterial gyrase. *Science Reports*.

[16] Llor C., Bjerrum L. (2014). Antimicrobial resistance: risk associated with antibiotic overuse and initiatives to reduce the problem. *Therapeutic Advances in Drug Safety*.

[17] Joya M., Aalemi A. K., Baryali A. T. (2022). Prevalence and antibiotic susceptibility of the common bacterial uropathogen among UTI patients in French Medical Institute for Children. *Infection and drug resistance*.

[18] Gninkoun J. C., Alassani A. S. C., Sagna Y., Djrolo J. (2018). Résistance bactérienne au cours des infections urinaires chez les patients diabétiques au CNHU-HKM de Cotonou, Bénin. *Journal de la Société de Biologie Clinique du Bénin*.

[19] Wójcik-Bojek U., Różalska B., Sadowska B. (2022). *Staphylococcus aureus*-a known opponent against host defense mechanisms and vaccine development-do we still have a chance to win?. *International Journal of Molecular Sciences*.

[20] Tong S. Y., Davis J. S., Eichenberger E., Holland T. L., Fowler V. G. (2015). *Staphylococcus aureus* infections: epidemiology, pathophysiology, clinical manifestations, and management. *Clinical Microbiology Reviews*.

[21] Martins K. B., Ferreira A. M., Pereira V. C., Pinheiro L., de Oliveira A., da Cunha M. L. R. S. (2019). *In Vitro* effects of antimicrobial agents on planktonic and biofilm forms of *Staphylococcus saprophyticus* isolated from patients with urinary tract infections. *Frontier in Microbiology*.

[22] Dumaru R., Baral R., Shrestha L. B. (2019). Study of biofilm formation and antibiotic resistance pattern of Gram-negative bacilli among the clinical isolates at BPKIHS, Dharan. *BMC Research Notes*.

[23] Pérez-Montarelo D., Viedma E., Larrosa N. (2018). Molecular epidemiology of *Staphylococcus aureus* bacteremia: association of molecular factors with the source of infection. *Frontier in Microbiology*.

[24] Schwartz D. (1960). La méthode statistique en médecine : les enquêtes éthiologiques. *Revue de Statistique appliquée*.

[25] Akpovi J., Perrin R. X., Alihonou E. (1998). Etude des facteurs de risque de prématurité à Cotonou. *Bénin Médical*.

[26] Riegel P., Archambaud M., Clavé D., Vergnaud M. (2006). *Bactérie de culture et d’identification difficiles*.

[27] Couto I., Pereira S., Miragaia M., Sanches I. S., de Lencastre H. (2001). Identification of clinical staphylococcal isolates from humans by internal transcribed spacer PCR. *Journal of Clinical Microbiology*.

[28] CASFM (2020). *Comité de l’antibiogramme de la Société Française de Microbiologie: Recommandations 2020 V.1.1*.

[29] Assouma F. F., Sina H., Adjobimey T. (2023). Susceptibility and virulence of *Enterobacteriaceae* isolated from urinary tract infections in Benin. *Microorganisms*.

[30] Socohou A., Sina H., Degbey C. (2021). Pathogenicity and molecular characterization of *Staphylococcus aureus* strains isolated from the hospital environment of CHU-Z Abomey-Calavi/Sô-Ava (Benin). *BioMed Research International*.

[31] Kırmusaoğlu S. (2019). The methods for detection of biofilm and screening antibiofilm activity of agents. *Antimicrobials, antibiotic resistance, antibiofilm strategies and activity methods*.

[32] Benaissa E., Belouad E., Mechal Y. (2021). Multidrug-resistant community-acquired urinary tract infections in a northern region of Morocco: epidemiology and risk factors. *Germs*.

[33] Rachid N. (2014). *Epidémiologie et résistance aux antibiotiques des bactéries isolées d’infection urinaires à l’hôpital militaire d’instruction Mohammed V de rabat*.

[34] Hailaji N. S. M., Ould Salema M. L., Ghaber S. M. (2016). La sensibilité aux antibiotiques des bactéries uropathogènes dans la ville de Nouakchott-Mauritanie. *Progrès en Urologie*.

[35] Amet Di Y. (2014). Bilan et profil de sensibilité aux antibiotiques des souches bactériennes isolées des infections du tractus urinaire de janvier 2003 à décembre 2013 dans le laboratoire d’analyses de biologie médicale Bio 24 à Dakar (Sénégal).

[36] Adeep M., Nima T., Kezang W., Tshokey T. (2016). A retrospective analysis of the etiologic agents and antibiotic susceptibility pattern of uropathogens isolated in the Jigme Dorji Wangchuck National Referral Hospital, Thimphu, Bhutan. *BMC Research Notes*.

[37] Benhiba I., Bouzekraoui T., Zahidi J. (2015). Épidémiologie et antibioresistance des infections urinaires à entérobactéries chez l’adulte dans le CHU de Marrakech et implications thérapeutiques. *Revue africaine d'urologie et d'andrologie*.

[38] Lazrak M., El Bardai A., Jaafour G., Kabbali S., Arrayhani N., Houssaini T. S. (2014). Profil de l’infection urinaire nosocomiale dans un service de néphrologie. *The Pan African Medical Journal*.

[39] Lacheheub L., Bendagha Y. (2016). *Les infections urinaires*.

[40] Rowe T. A., Juthani-Mehta M. (2013). Urinary tract infection in older adults. *Aging health*.

[41] Benyagoub E., Benyagoub E., Berbaoui H., Rahmani C., Benyoucef L. (2013). Identification and study of the emergence of antibiotic resistance of microorganisms responsible for urinary tract infections in Bechar (Algeria). *Science en Liberté*.

[42] Gajdács M., Ábrók M., Lázár A., Burián K. (2020). Increasing relevance of Gram-positive cocci in urinary tract infections: a 10-year analysis of their prevalence and resistance trends. *Scientific Reports*.

[43] von Eiff C., Peters G., Heilmann C. (2002). Pathogenesis of infections due to coagulasenegative staphylococci. *The Lancet Infectious Diseases*.

[44] Ait Miloud K. (2011). *L’infection urinaire: expérience du laboratoire de microbiologie de l’hôpital des spécialités de Rabat*.

[45] Dikoumba A., Onanga R., Nguema P. (2021). Phenotipic prevalence of antibiotic resistance in Gabon. *Open Journal of Medical Microbiology*.

[46] Naorem R. S., Urban P., Goswami G., Fekete C. (2020). Characterization of methicillin-resistant *Staphylococcus aureus* through genomics approach. *3 Biotech*.

[47] Hachemi A., Zenia S., Denia M. F., Guessoum M., Hachemi M. M., Ait-Oudhia K. (2019). Epidemiological study of sausage in Algeria: prevalence, quality assessment, and antibiotic resistance of *Staphylococcus aureus* isolates and the risk factors associated with consumer habits affecting foodborne poisoning. *Veterinary World*.

[48] Shenkman B., Rubinstein E., Cheung A. L. (2001). Adherence properties of *Staphylococcus aureus* under static and flow conditions: roles ofagrandsarLoci, platelets, and plasma ligands. *Infection and Immunity*.

[49] Fitzpatrick F., Humphreys H., Smyth E., Kennedy C. A., O'Gara J. P. (2002). Environmental regulation of biofilm formation in intensive care unit isolates of *Staphylococcus epidermidis*. *Journal of Hospital Infection*.

[50] Tanih N. F., Sekwadi E., Ndip R. N., Bessong P. O. (2015). Detection of pathogenic *Escherichia coli* and *Staphylococcus aureus* from cattle and pigs slaughtered in abattoirs in Vhembe District, South Africa. *The Scientific World Journal*.

[51] Rocchetti T. T., Martins K. B., Martins P. Y. F. (2018). Detection of the *mec* A gene and identification of *Staphylococcus* directly from blood culture bottles by multiplex polymerase chain reaction. *Brazilian Journal of Infectious Diseases*.

